# Choline supplementation protects against sepsis-induced lung injury, potentially through suppression of Prtn3-associated monocyte activation

**DOI:** 10.3389/fphar.2026.1839871

**Published:** 2026-06-18

**Authors:** Li-Ming Xu, Wei-Can Chen, Zhen-Dong Sun, Li-Hong Zhang, Yi-Bin Liu, Xiao-Ting Luo, Yan Chen, Lin Lin, He-Fan He

**Affiliations:** Department of Anesthesiology, The Second Affiliated Hospital of Fujian Medical University, Quanzhou, China

**Keywords:** choline, monocyte/macrophage activation, multi-omics, PRTN3, sepsis-induced lung injury

## Abstract

**Objective:**

Choline supplementation has been implicated in the regulation of inflammation and immune responses. This study aimed to investigate the protective effects of choline supplementation in sepsis-induced lung injury (SLI) and to elucidate the underlying mechanisms.

**Methods:**

SLI was induced in mice via cecal ligation and puncture (CLP). Serum choline levels were measured, and choline supplementation effects were evaluated *in vivo*. Integrated transcriptomic and proteomic analyses identified potential targets and pathways. Public single-cell RNA sequencing data determined the cellular distribution of candidate genes. *In vitro* assays in THP-1 cells were performed to validate the role of key targets.

**Results:**

Compared with Sham controls, SLI mice exhibited significantly decreased serum choline levels. The SLI mouse model exhibited typical pathological features, including pulmonary hemorrhage, interstitial edema, alveolar wall thickening, elevated inflammatory cytokines, and increased lung injury scores, all of which were significantly alleviated by choline supplementation. Multi-omics analyses identified Prtn3 as a potential target associated with the protective effects of choline. Single-cell analysis suggested that Prtn3 is predominantly expressed in monocyte/macrophage populations. *In vitro*, choline treatment attenuated LPS-induced inflammatory responses and chemotactic activity in THP-1 cells, accompanied by reduced expression of IL-1β, IL-6, TNF-α, Prtn3 and NF-κB pathway proteins. Prtn3 overexpression partially reversed the inhibitory effects of choline on cytokine production, monocyte migration, and NF-κB pathway activation. Conversely, pharmacological inhibition of Prtn3 by sivelestat suppressed inflammatory cytokine expression and attenuated NF-κB signaling under LPS-stimulated conditions.

**Conclusion:**

Choline supplementation protects against SLI by suppressing monocyte inflammatory activation, at least in part through inhibition of the Prtn3-dependent NF-κB signaling pathway. These findings suggest that targeting the choline–Prtn3–NF-κB axis may represent a potential therapeutic strategy for sepsis-associated lung injury.

## Introduction

Sepsis is a life-threatening organ dysfunction caused by a dysregulated host response to infection and remains a major global health burden with high mortality rates. The mortality of septic shock is approximately 38% in Europe and North America (Vincent et al., 2019), while in China, sepsis affects about 25.5% of intensive care unit (ICU) patients and is associated with a mortality rate approaching 40% (Lei et al., 2022). The lung is one of the earliest and most susceptible target organs during sepsis-induced multiple organ dysfunction (Sun et al., 2023). It has been reported that 25%–50% of septic patients develop sepsis-induced acute lung injury (SLI), which is characterized by excessive pulmonary inflammation, increased vascular permeability, alveolar damage, and respiratory failure (Qiao et al., 2024). Given that uncontrolled inflammation is a central driver of SLI, strategies aimed at modulating inflammatory responses and promoting lung repair are of considerable therapeutic interest.

Choline is an essential nutrient in mammals and a critical component of acetylcholine and phosphatidylcholine, playing indispensable roles in metabolism and the physiological functions of the heart, brain, liver, and skeletal muscle (Zeisel and da Costa, 2009). Although choline can be obtained from dietary sources such as meat, milk, fish, eggs, and legumes (Wiedeman et al., 2018; Lewis et al., 2015), insufficient intake has been associated with tissue injury and inflammatory responses (Zeisel and da Costa, 2009; Buchman et al., 2001). Increasing evidence suggests that choline supplementation exerts anti-inflammatory and immunomodulatory effects. For instance, choline chloride has been shown to alleviate allergic airway disease by reducing airway inflammation, hyperresponsiveness, and oxidative stress (Bansal et al., 2022). Clinical studies have also demonstrated that choline supplementation can significantly decrease inflammatory cytokine levels, highlighting its therapeutic potential in inflammatory airway diseases such as asthma (Mehta et al., 2010; Detopoulou et al., 2008). Moreover, choline supplementation has been reported to upregulate anti-inflammatory cytokines while suppressing pro-inflammatory mediators, including TNF-α, IL-1β, and NF-κB (Koca et al., 2008; Wu et al., 2016; Zhao et al., 2016).

Despite these insights, the role of choline in sepsis remains incompletely understood. A recent study showed that choline supplementation improved renal function in septic mice but did not significantly affect survival, systemic inflammation, or metabolic pathways (Hasson et al., 2022), suggesting that its effects may be organ-specific or context-dependent. Whether choline can similarly mitigate SLI has yet to be determined.

In this study, we systematically investigated the effects of choline supplementation on SLI. We employed transcriptomic and proteomic analyses to comprehensively characterize the molecular alterations associated with choline treatment. Furthermore, single-cell RNA sequencing was conducted to delineate the cellular heterogeneity and underlying mechanisms by which choline exerts its protective effects in the septic lung. This multi-omics approach provides a mechanistic framework for evaluating choline as a potential therapeutic strategy against SLI.

## Method and material

### Animal experiment

Male C57BL/6 mice (8–10 weeks old, 25–30 g) were obtained from Beijing Vital River Laboratory Animal Technology (Beijing, China). All mice were housed at four per cage in a temperature-controlled room under a 12-h light–dark cycle. Mice were used in two independent experimental sets. In the first set, mice were randomly divided into two groups: the Sham group and the SLI group (n = 6 for each group). In the second set, mice were randomly assigned to four groups: Sham group, SLI group, SLI + low-dose choline group (Choline_L, 80 mg/kg), and SLI + high-dose choline group (Choline_H, 100 mg/kg) (n = 6 for each group). The sample size was selected based on previous studies using the CLP model to detect biologically meaningful differences in lung injury and inflammatory markers (Qassam et al., 2025; Nullens et al., 2018). SLI model was established using the cecal ligation and puncture (CLP) procedure. Choline chloride (GLPBIO, Montclair, CA, United States) was dissolved in sterile normal saline and administered intraperitoneally to SLI mice at doses of 80 or 100 mg/kg. Choline was administered at 2, 6, and 12 h after surgery. Dose timing and concentrations were selected based on earlier studies demonstrating effective elevation of plasma choline levels (Hasson et al., 2022; Ilcol et al., 2002). Twenty-four hours after CLP, mice were euthanized, and serum and lung tissues were collected for further analysis. All animal experiments were conducted in accordance with the National Institutes of Health (NIH) guidelines and were approved by the Laboratory Animal Management and Welfare Ethics Committee of the Second Affiliated Hospital of Fujian Medical University (Approval No. 2025257; approved on 1 May 2025).

### Cecal ligation and puncture (CLP) model

A mouse model of SLI was established using CLP procedure. Briefly, mice were anesthetized by intraperitoneal injection of pentobarbital sodium (1%, 50 mg/kg; Sigma, United States). A midline abdominal incision (∼1 cm) was made to expose the cecum, which was carefully isolated, ligated, and punctured with a 20-gauge needle. A small amount of fecal content was gently extruded into the peritoneal cavity. The cecum was then returned to the abdominal cavity, and the incision was closed in layers. Immediately after surgery, mice received subcutaneous fluid resuscitation with prewarmed saline (25 mL/kg). To ensure animal welfare, mice were administered analgesia with buprenorphine (0.05 mg/kg, subcutaneously) immediately after surgery. Mice were closely monitored for 24 h postoperatively, including assessment of activity, diarrhea, lethargy, fur condition, and eye appearance, to evaluate general health and severity of sepsis. A standardized murine sepsis score (MSS), incorporating these parameters, was applied to quantify clinical severity, ensuring consistency and reproducibility of the model (Shrum et al., 2014). Sham-operated mice underwent the same procedure without ligation or puncture. All mice had free access to food and water and were monitored postoperatively.

### H&E staining and lung injury score evaluation

Lung samples were first fixed in 10% neutral buffered formalin, followed by paraffin embedding and sectioning into approximately 4 µm slices. The resulting sections were then subjected to hematoxylin and eosin (H&E) staining (Servicebio, Wuhan, China) and examined using light microscopy. Lung injury severity was evaluated based on established guidelines (Li Y. et al., 2020).

### Determination of protein content in bronchoalveolar lavage fluid (BALF)

BALF was collected from the left lung to allow separate use of the contralateral lung lobes for histological, molecular, and wet-to-dry weight analyses. Briefly, mice were euthanized, and the trachea was exposed through a midline cervical incision. The right main bronchus was ligated to prevent lavage fluid from entering the right lung. The left bronchus was then cannulated with a fine needle, and the left lung was lavaged five times with 0.5 mL sterile PBS. Each aliquot was gently instilled and aspirated after a 1-min dwell time. The recovered BALF was pooled and centrifuged at 1,500 rpm for 10 min at 4 °C. The supernatant was stored at −20 °C until protein quantification. BALF protein concentration was measured using a BCA assay kit (Beyotime, China).

### Lung wet-to-dry weight ratio measurement

Pulmonary edema was assessed by measuring the wet-to-dry (W/D) weight ratio of the right lung. After BALF collection from the left lung, the right lung was carefully excised, gently rinsed with PBS to remove surface blood, blotted dry, and immediately weighed to obtain the wet weight. The tissue was then dried in an incubator at 70 °C for 48 h until a stable dry weight was reached. The dry weight was recorded, and the W/D ratio was calculated as wet weight divided by dry weight.

### Immunofluorescence staining of F4/80 in lung tissue

Paraffin-embedded lung tissue sections were deparaffinized in xylene and rehydrated through a graded ethanol series. Antigen retrieval was performed by heating the sections in citrate buffer, followed by cooling to room temperature. After washing with PBS, sections were permeabilized with 0.1% Triton X-100 and blocked with 5% bovine serum albumin for 1 h at room temperature. The sections were then incubated overnight at 4 °C with a primary antibody against F4/80. After three washes with PBS, the sections were incubated with a fluorescence-conjugated secondary antibody for 1 h at room temperature in the dark. Nuclei were counterstained with DAPI, and the sections were mounted with antifade mounting medium. Fluorescence images were acquired using a fluorescence microscope under identical exposure settings. The F4/80-positive fluorescence area or integrated fluorescence intensity was quantified using ImageJ software.

### Cell culture and transfection

The human monocytic leukemia cell line THP-1 was acquired from the American Type Culture Collection (ATCC, located in Manassas, VA, United States). THP-1 cells were grown in RPMI 1640 medium (Servicebio, Wuhan, China). Each medium was enriched with 10% fetal bovine serum (FBS; Gibco, Waltham, MA, United States) and 1% penicillin/streptomycin (P/S; Biosharp, Hefei, China). Cells were incubated at 37 °C in a humidified atmosphere containing 5% CO_2_.

### Lentivirus transfection

Lentiviral particles for Prtn3 overexpression (LV-Prtn3) and the corresponding negative control vector (LV-NC) were purchased from Genechem Corporation. The LV-Prtn3 vector carried a GFP-tagged Prtn3 construct, whereas LV-NC contained the empty vector backbone. THP-1 cells were seeded in 12-well plates and transduced with LV-Prtn3 or LV-NC for 72 h, followed by selection with puromycin (2 μg/mL) to establish stable cell lines. Cells were maintained under puromycin selection for 3 weeks. The overexpression efficiency of Prtn3 was confirmed by Western blot.

### Cell viability assay

The cell viability was assessed using a Cell Counting Kit-8 (CCK-8) assay. Briefly, 10 μL of CCK-8 reagent was supplemented into each well of a 96-well plate followed by incubation for 1 hour at 37 °C. Absorbance was then measured at a wavelength of 450 nm in accordance with the manufacturer’s protocol.

### Cell migration assay

Cell migration was assessed using Transwell chambers (24-well plates, 8 μm pore size; Corning, NY, United States). THP-1 cells (1 × 10^5^) were suspended in 200 μL of serum-free RPMI 1640 medium and added to the upper chamber, while the lower chamber was filled with 750 μL of RPMI 1640 containing 10% fetal bovine serum (FBS) as a chemoattractant. Cells were incubated at 37 °C in a humidified 5% CO_2_ atmosphere for 24 h. After incubation, non-migrated cells on the upper surface of the membrane were gently removed with a cotton swab. Migrated cells on the underside were fixed with 4% paraformaldehyde for 15 min, stained with 0.1% crystal violet for 20 min, rinsed with PBS, and imaged under a microscope at 200× magnification. Five random fields per insert were counted, and all experiments were performed in triplicate.

### Quantitative real-time PCR

Total RNA was isolated with the RNeasy Mini Kit (Cat#: 74104, Qiagen) according to the manufacturer’s instructions. Complementary DNA (cDNA) was synthesized using the GoScript™ Reverse Transcription System (Cat#: A5000, Promega). Gene expression levels were quantified by real-time PCR with SYBR GreenER™ qPCR SuperMix Universal (Cat#: 1176202K, Invitrogen), and the 2^−ΔΔCT^ method was applied to analyze the results, using GAPDH as the endogenous reference. The primer sequences used are listed in Table 1.

**TABLE 1:** The primer sequence of target genes.

IL1B-F (mus)	GAA​ATG​CCA​CCT​TTT​GAC​AGT​G
IL1B-R (mus)	TGG​ATG​CTC​TCA​TCA​GGA​CAG
IL6-F (mus)	CTG​CAA​GAG​ACT​TCC​ATC​CAG
IL6-R (mus)	AGT​GGT​ATA​GAC​AGG​TCT​GTT​GG
TNF-a-F (mus)	GCC​GAT​GGG​TTG​TAC​CTT​GT
TNF-a-R (mus)	TCT​TGA​CGG​CAG​AGA​GGA​GG
IL1B-F (homo)	GCT​TAT​TAC​AGT​GGC​AAT​GAG​GAT
IL1B-R (homo)	TAG​TGG​TGG​TCG​GAG​ATT​CG
Il6-F (homo)	AAG​CCA​GAG​CTG​TGC​AGA​TG
Il6-R (homo)	CTG​GCA​TTT​GTG​GTT​GGG​TC
TNFα-F (homo)	TCC​TCT​CTG​CCA​TCA​AGA​GC
TNFα-R (homo)	AGT​AGA​CCT​GCC​CAG​ACT​CG
Prtn3-F (homo)	GTG​GCT​CAG​GTG​TTT​CTG​AAC
Prtn3-R (homo)	GAC​GAA​AGT​GCA​AAT​GTT​ATG​T

### Western blot

Protein lysates were prepared from lung tissues or cultured cells using RIPA buffer, and protein concentrations were determined with a BCA assay kit (P0011, Beyotime, China). Aliquots containing 50 µg of protein from each sample were separated by 10% SDS-PAGE and subsequently transferred onto PVDF membranes. After blocking with 5% non-fat dry milk, the membranes were incubated with specific primary antibodies overnight at 4 °C. The following primary antibodies were used: GAPDH (1:10,000, AF7021, Affinity) and Prtn3 (1:2000, ER1706-25, HuaBio). Following primary antibody incubation, the blots were treated with an HRP-conjugated secondary antibody (1:10,000, Proteintech) for 1 hour at room temperature. Protein bands were visualized using an ECL detection kit (Beyotime) and analyzed with ImageJ software. After background subtraction, the integrated density of each target band was measured. Target protein expression was normalized to GAPDH from the same lane.

### Immunofluorescence staining

THP-1 cells from each treatment group were collected, washed with PBS, and seeded onto poly-L-lysine-coated glass coverslips to allow cell attachment. Cells were fixed with 4% paraformaldehyde for 15 min at room temperature, permeabilized with 0.1% Triton X-100 for 10 min, and blocked with 5% bovine serum albumin for 1 h. The cells were then incubated overnight at 4 °C with primary antibodies against p-NF-κB and p-IKKβ. After washing with PBS, cells were incubated with fluorescence-conjugated secondary antibodies for 1 h at room temperature in the dark. Nuclei were counterstained with DAPI, and coverslips were mounted with antifade mounting medium. Fluorescence images were acquired using a fluorescence microscope under identical exposure settings. The fluorescence intensity was quantified using ImageJ software.

### RNA-seq and data analysis

Total RNA was isolated and purified from lung tissues of septic mice using the TRIzol method (Invitrogen, CA, United States). For sequencing, six independent biological replicates per group (n = 6) were used. RNA concentration and purity were assessed using a NanoDrop 2000 spectrophotometer (Thermo Scientific, Waltham, MA, United States), and RNA integrity was evaluated using the Agilent 5,400 Fragment Analyzer system (Agilent Technologies, CA, United States). Poly(A)+ RNA was enriched from 1 μg of total RNA using Dynabeads Oligo (dT) (Thermo Fisher Scientific, CA, United States) through two rounds of purification. First-strand cDNA was synthesized using random hexamer primers and M-MuLV Reverse Transcriptase (RNase H−). Subsequently, second-strand cDNA synthesis was performed using DNA Polymerase I and RNase H. The resulting double-stranded cDNA underwent end repair to generate blunt ends via exonuclease and polymerase activities, followed by enzyme removal. After adenylation of the 3′ends, Illumina paired-end (PE) adapter oligonucleotides were ligated to the cDNA fragments. Fragments of approximately 350 bp were selected and purified using the AMPure XP system (Beckman Coulter, Beverly, CA, United States). cDNA fragments with adapters ligated to both ends were enriched by PCR amplification using the Illumina PCR Primer Cocktail with 15 cycles. The PCR products were purified using the AMPure XP system and quantified using the Agilent High Sensitivity DNA assay on the 5,400 Fragment Analyzer system (Agilent Technologies, CA, USA). The prepared libraries were sequenced on the Illumina NovaSeq 6,000 platform, generating 150 bp paired-end reads (Shanghai Biotree Biotech Co., Ltd.). Differentially expressed genes (DEGs) were identified using |log_2_ (fold change)| > 1.0 and an adjusted P-value < 0.05, with multiple testing correction using the Benjamini–Hochberg method. DEG distributions were visualized using volcano plots. Functional enrichment and pathway analyses were performed using Gene Ontology (GO) and Kyoto Encyclopedia of Genes and Genomes (KEGG) databases.

### Proteomics

For proteomic analysis, three independent biological replicates per group (n = 3) were used. For each sample, 500 ng of total peptides were separated and analyzed using a nano-UPLC system (Vanquish Neo, Thermo Scientific) coupled to an Astral mass spectrometer (Thermo Scientific) equipped with a nano-electrospray ionization source. Peptide separation was performed on a reversed-phase column (EASY-Spray™ HPLC column, 150 mm × 15 cm, Thermo Scientific, United States). The mobile phases consisted of (A) water with 0.1% formic acid (FA) and (B) 80% acetonitrile (ACN) with 0.1% FA. Peptides were separated using an 8 min gradient. Data-independent acquisition (DIA) was conducted in positive ion mode. MS1 spectra were acquired in profile mode using the Orbitrap analyzer at a resolution of 240,000 over an *m/z* range of 380–980. MS2 spectra were acquired over an *m/z* range of 150–2000 using higher-energy collisional dissociation (HCD) with a normalized collision energy (NCE) of 25% and an isolation window of 2 *m/z*. Raw MS data files were processed using DIA-NN software (version 1.8.1). Spectra were searched against a species-specific UniProt FASTA database (*Mus musculus*, UniProt ID: 10,090, reviewed, 2024 version). Carbamidomethylation of cysteine residues [C] was set as a fixed modification, while oxidation of methionine (M) and acetylation at the protein N-terminus were specified as variable modifications. Trypsin was used as the proteolytic enzyme, allowing up to two missed cleavages. The false discovery rate (FDR) was set to 1% at both peptide-spectrum match (PSM) and peptide levels. Peptide identification was performed with a precursor mass tolerance of 20 ppm and a fragment mass tolerance of 20 ppm. All other parameters were set to default values.

### Single-cell data processing and clustering

Publicly available scRNA-seq data (GSE207651) were retrieved from the GEO database and used as an independent validation dataset to assess the expression patterns of candidate biomarkers in septic lungs. Data were processed using Seurat (v4.1.0) in R. Cells were filtered based on the following quality control criteria: cells with nFeature_RNA < 300 or > 4,000, nCount_RNA > 20,000, or Percent.mt > 8% were excluded to remove low-quality, stressed, or potentially multiplet cells. After filtering, the top 3,000 highly variable genes for each sample were identified using variance stabilization transformation (vst). These genes were scaled via the ScaleData function, and principal component analysis (PCA) was applied for dimensionality reduction using RunPCA. Thirty principal components were selected to cluster cells into 13 populations with the FindNeighbors and FindClusters functions, and UMAP (RunUMAP) was used for visualization. Expression patterns of candidate biomarkers were visualized using FeaturePlot, displaying gene expression levels across cells in the UMAP embedding. To quantify expression across cell types, the average expression per cluster was calculated and plotted as a bar chart. The proportion of each cell type in control and sepsis samples was also calculated to assess population shifts.

### Statistical analysis

Statistical analyses were performed using SPSS software (version 23.0) and GraphPad Prism (version 8.0). Data are presented as mean ± standard deviation (SD). The number of biological replicates (n) for each experiment is indicated in the corresponding figure legends. For animal experiments, each biological replicate represents one independent mouse. For *in vitro* experiments, biological replicates represent independent cell culture experiments performed on separate occasions. Prior to analysis, data were assessed for normality and homogeneity of variance. For comparisons among multiple groups, one-way analysis of variance (ANOVA) followed by Tukey’s *post hoc* test was applied. For comparisons between two groups, an unpaired Student’s t-test was used. For non-normally distributed data, two-group comparisons were analyzed using the Mann–Whitney U test, whereas comparisons among more than two groups were performed using the Kruskal–Wallis test followed by Dunn’s *post hoc* test. A two-tailed *P* value < 0.05 was considered statistically significant. Sample sizes were selected based on our previous studies, published literature, and pilot experiments to ensure reproducibility and biological consistency.

## Results

### The serum level of choline was decreased in SLI mice

**FIGURE 1:**
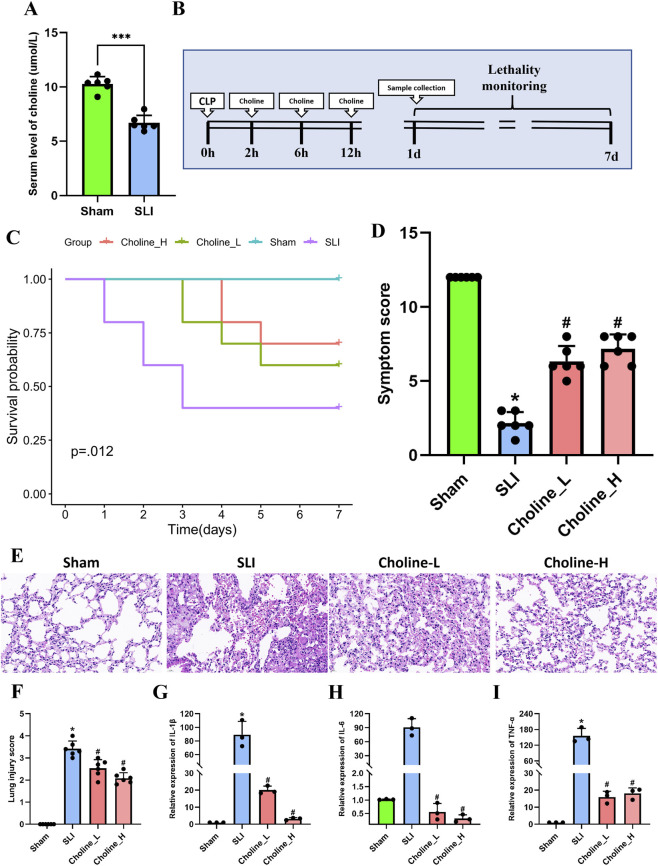
Effects of choline on survival and lung injury in septic mice. **(A)** Serum choline levels in SLI mice and Sham controls. ***P < 0.001 vs. Sham group, n = 6 for each group. **(B)** Schematic illustration of the experimental design. Mice underwent Sham or CLP surgery on day 0, followed by intraperitoneal injection of choline or PBS at 2, 6, and 12 h post-surgery. **(C)** Kaplan–Meier survival curves of Sham, SLI, and choline-treated mice over 7 days. n = 6 for each group. **(D)** Sepsis severity scores in each group. n = 6 for each group. **(E)** Representative H&E-stained lung sections from indicated groups. 40✕. **(F)** Quantification of lung injury scores. n = 6 for each group. **(G–I)** Relative mRNA expression levels of IL6, TNF-α, and IL-1β in lung tissues. n = 3 for each group. Data are presented as mean ± SD. *P < 0.05 vs. Sham; #P < 0.05 vs. SLI group.

Serum choline levels were first measured to assess potential alterations associated with SLI. Compared with Sham controls, SLI mice exhibited significantly decreased serum choline levels (Figure 1A).

### Choline supplementation improves survival and attenuates lung injury and pulmonary inflammation in septic mice

Given the reduction in serum choline observed in SLI mice, we next examined whether choline supplementation could confer protection in SLI. A CLP model was used to induce SLI in mice (Figure 1B). Choline was administered by intraperitoneal injection at a dose of low dose 80 mg/kg and high dose 100 mg/kg. Survival analysis showed that the 7-day survival rate was markedly reduced in SLI mice compared with Sham mice, whereas choline treatment improved survival following CLP challenge (Figure 1C). In parallel, sepsis severity scores were significantly increased in SLI mice and were ameliorated by choline treatment (Figure 1D). We next evaluated the effect of choline supplementation on lung injury in the CLP-induced SLI model. Histopathological examination by H&E staining showed that CLP caused marked lung injury, characterized by pulmonary hemorrhage, interstitial edema, inflammatory cell infiltration, alveolar wall thickening, elevated BALF protein concentration, whereas these pathological changes were noticeably attenuated in choline-treated mice (Figures 1E,F; Supplementary File 2; Supplementary Figure S1). Consistently, lung injury scores were significantly increased in SLI mice and were reduced following choline treatment. To further assess pulmonary inflammation, the expression of inflammatory cytokines in lung tissue was measured. The mRNA levels of IL-6, TNF-α, and IL-1β were significantly increased in the lungs of SLI mice but were reduced by choline treatment (Figures 1G–I). These data demonstrate that choline supplementation alleviates CLP-induced lung injury and pulmonary inflammatory responses *in vivo*.

### Transcriptomic profiling identifies genes reversed by choline treatment in SLI lung tissue

**FIGURE 2:**
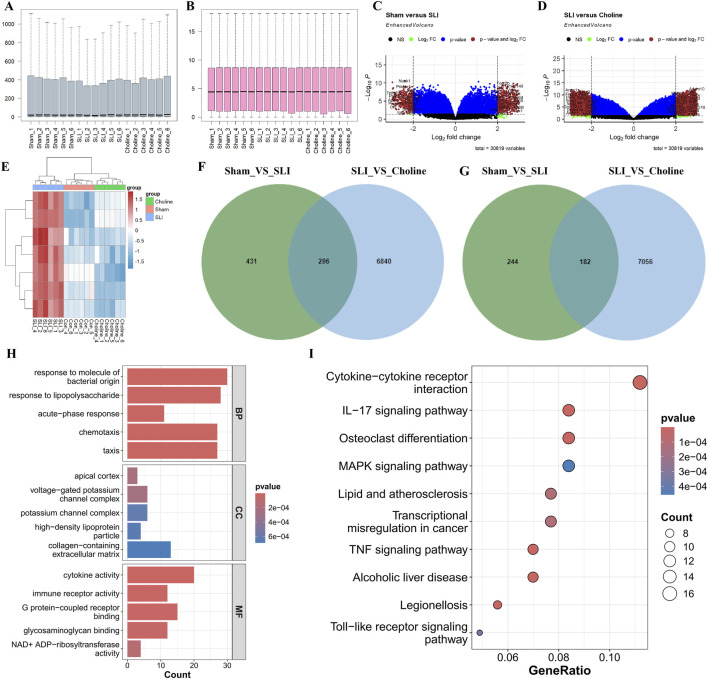
Transcriptomic analysis of Sham, SLI, and choline-treated groups. **(A,B)** Distribution of mRNA expression across samples before **(A)** and after **(B)** batch correction (n = 6 for group). **(C)** Volcano plot of differentially expressed genes (DEGs) between Sham and SLI groups. **(D)** Volcano plot of DEGs between SLI and choline-treated groups. **(E)** Heatmap. **(F,G)** Venn diagrams showing upregulated **(F)** and downregulated **(G)** DEGs in SLI compared with Sham and choline groups. **(H)** Gene Ontology (GO) enrichment analysis. **(I)** KEGG pathway enrichment analysis.

To explore the molecular basis underlying the protective effect of choline, transcriptomic profiling was performed using lung tissue samples from Sham, SLI, and choline-treated SLI mice. Global gene expression distributions were comparable across samples, indicating good overall data quality (Figures 2A,B). Differential expression analysis identified 1,153 genes that were significantly dysregulated in the SLI group compared with the Sham group, using the criteria of |log2 fold change| ≥ 2 and *P* < 0.05 (Figure 2C). Compared with the SLI group, 14,374 DEGs were identified in the choline-treated group applying the same thresholds (Figure 2D). To assess the restorative effect of choline, we defined reversed genes as genes that were significantly dysregulated in the SLI group relative to the Sham group and showed a significant change in the opposite direction in the choline-treated group relative to the SLI group. Based on this definition, 478 genes were identified as reversed genes (Figures 2E–G; Supplementary File 1; Supplementary Table S1). These findings suggest that choline partially restores the altered transcriptional program associated with SLI.

### Functional enrichment analysis of reversed genes implicates inflammatory and immune regulatory pathways

To gain insight into the biological significance of the reversed genes, Gene Ontology (GO) and Kyoto Encyclopedia of Genes and Genomes (KEGG) enrichment analyses were performed. All enrichment analyses were corrected for multiple testing, and significantly enriched terms were selected based on q-value < 0.05 to control for false discovery rate (FDR). GO biological process analysis showed that the reversed genes were significantly enriched in pathways related to leukocyte activation, inflammatory responses, tumor necrosis factor production, B-cell differentiation, and Toll-like receptor signaling (Figure 2H; Supplementary File 1; Supplementary Table S2). KEGG pathway analysis further revealed enrichment in pathways including lipid and atherosclerosis, Toll-like receptor signaling, MAPK signaling, and transcriptional misregulation-related pathways (Figure 2I; Supplementary File 1; Supplementary Table S3). These results suggest that choline treatment may exert protective effects in SLI by modulating inflammatory and immune signaling pathways.

### Proteomic analysis identifies proteins reversed by choline treatment in SLI mice

**FIGURE 3:**
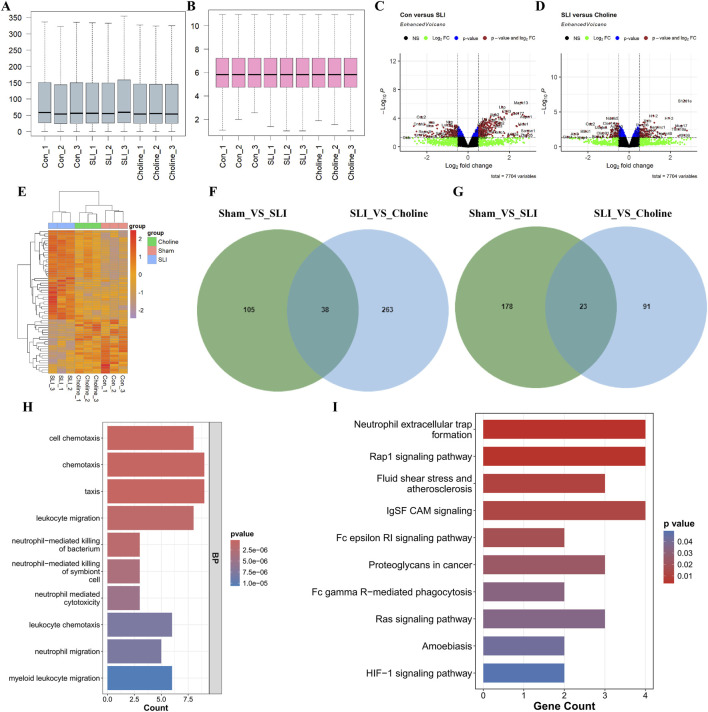
Proteomic analysis of Sham, SLI, and choline-treated groups. **(A,B)** Distribution of protein expression across samples before **(A)** and after **(B)** batch correction (n = 3 for group). **(C)** Volcano plot of differentially expressed proteins (DEPs) between Sham and SLI groups. **(D)** Volcano plot of DEPs between SLI and choline-treated groups. **(E)** Heatmap. **(F,G)** Venn diagrams showing upregulated **(F)** and downregulated **(G)** DEPs in SLI compared with Sham and choline groups. **(H)** Gene Ontology (GO) enrichment analysis. **(I)** KEGG pathway enrichment analysis.

To complement the transcriptomic analysis, quantitative proteomic profiling was performed using a directDIA workflow. Global protein expression distributions were comparable across samples, indicating good overall data quality (Figures 3A,B). Compared with the Sham group, 502 differentially expressed proteins (DEPs) were identified in the SLI group, including 301 upregulated and 201 downregulated proteins, based on |log2 fold change| > 0.5 and P ≤ 0.05 (Figure 3C). Compared with the SLI group, 257 DEPs were identified in the choline-treated group, including 143 upregulated and 114 downregulated proteins, applying the same thresholds (Figure 3D). Reversed proteins were defined as those that were dysregulated in SLI versus Sham and showed a significant change in the opposite direction after choline treatment. Using this definition, 61 proteins were identified as reversed proteins (Figures 3E–G; Supplementary File 1; Supplementary Table S4). These results indicate that choline treatment partially restores proteomic alterations induced by SLI.

### Functional enrichment of reversed proteins suggests regulation of leukocyte migration and inflammatory responses

GO enrichment analysis of the reversed proteins showed significant enrichment in biological processes related to chemotaxis, epithelial cell migration, granulocyte chemotaxis, response to molecules of bacterial origin, regulation of interleukin-1 production, and cell killing (Figure 3H; Supplementary File 1; Supplementary Table S5). KEGG pathway analysis indicated that these reversed proteins were associated with neutrophil extracellular trap formation, Rap1 signaling, Fc epsilon RI signaling, and fluid shear stress-related pathways (Figure 3I; Supplementary File 1; Supplementary Table S6). To identify common biological themes across omics layers, we compared enrichment results from the transcriptomic and proteomic datasets. GO analysis revealed 48 biological processes shared by reversed genes and reversed proteins, including chemotaxis, leukocyte migration, myeloid leukocyte activation, neutrophil migration, and neutrophil chemotaxis. KEGG analysis identified three pathways common to both datasets, including amoebiasis, fluid shear stress and atherosclerosis, and Ras signaling (Supplementary File 1; Supplementary Tables S2, S3, S5, S6). Collectively, the shared pathways between transcriptomic and proteomic datasets indicate that choline consistently regulates inflammatory and immune processes, providing a mechanistic link between gene and protein-level alterations and suggesting that choline may modulate SLI progression, at least in part, by controlling leukocyte recruitment and inflammatory cell migration.

### Integrated transcriptomic and proteomic analysis identifies Prtn3 as a candidate hub molecule in SLI

**FIGURE 4:**
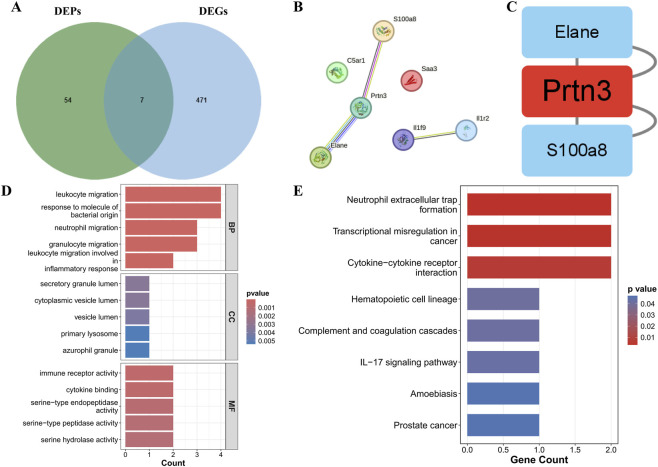
Integrated analysis of transcriptomics and proteomics. **(A)** Venn diagram showing overlapping targets between reversed genes and proteins. **(B)** PPI network of overlapping targets. **(C)** PPI network highlighting top-ranked targets based on degree score. **(D)** GO enrichment analysis of overlapping targets. **(E)** KEGG pathway enrichment analysis.

To identify key molecular mediators associated with choline treatment, transcriptomic and proteomic datasets were integrated, revealing seven overlapping genes/proteins (Figure 4A). Protein-protein interaction (PPI) analysis of these overlapping candidates indicated that Prtn3 exhibited the highest network connectivity, suggesting it may serve as a hub molecule mediating the protective effects of choline in SLI (Figures 4B,C). Functional enrichment analysis of the overlapping genes/proteins demonstrated significant involvement in neutrophil migration, granulocyte migration, leukocyte migration involved in inflammatory response, and myeloid leukocyte migration (Figure 4D; Supplementary File 1; Supplementary Table S7). KEGG analysis suggested that these molecules were involved in pathways such as neutrophil extracellular trap formation and cytokine-cytokine receptor interaction (Figure 4E; Supplementary File 1; Supplementary Table S8), highlighting the consistent regulation of immune and inflammatory processes by choline.

### Single-cell transcriptomic analysis localizes Prtn3 expression predominantly to monocytes

**FIGURE 5:**
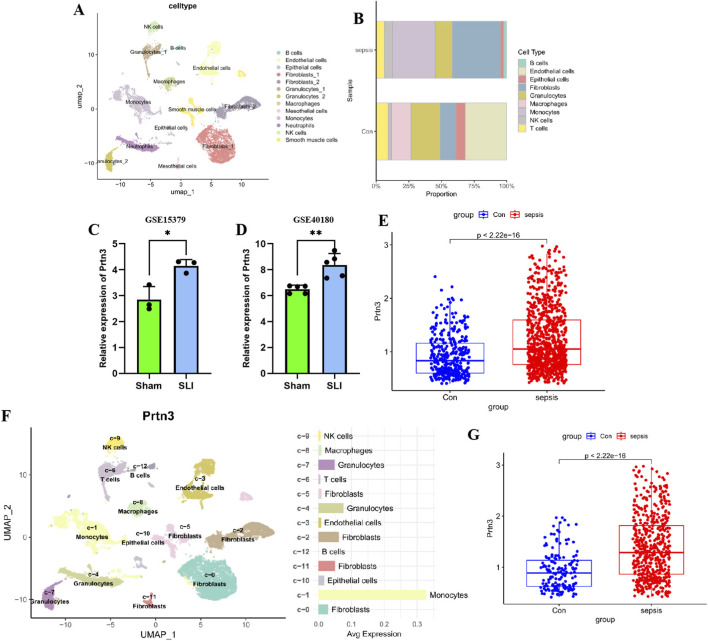
Expression profile of Prtn3 in lung tissues. **(A)** UMAP visualization of cell clusters in the scRNA-seq dataset (GSE207651). **(B)** Proportions of different cell populations. **(C–E)** Boxplots showing Prtn3 expression levels in GSE15379 **(C)**, GSE40180 **(D)**, and GSE207651 **(E)**. **(F)** UMAP plot showing Prtn3 expression across cell clusters. **(G)** Prtn3 expression in pulmonary monocytes. Statistical comparisons were performed using the Wilcoxon rank-sum test.

To further define the cellular source of the candidate hub gene Prtn3, we analyzed the public single-cell RNA-seq dataset GSE207651. After quality control (Supplementary File 2; Supplementary Figure S2), a total of 16,324 cells were retained and grouped into 13 clusters (Supplementary File 2; Supplementary Figure S3). Based on established marker genes, these clusters were annotated as fibroblasts, monocytes, endothelial cells, neutrophils, smooth muscle cells, macrophages, NK cells, epithelial cells, mesothelial cells, and B cells (Figure 5A; Supplementary File 2; Supplementary Figure S4). Comparison of cell composition between control and SLI samples revealed shifts in several immune and stromal cell populations, particularly fibroblasts, neutrophils, monocytes, and granulocyte-related clusters (Figure 5B). To further examine Prtn3 expression, we analyzed external datasets GSE15379 and GSE40180 and found that Prtn3 expression was increased in sepsis- or lung injury-related conditions (Figures 5C,D). This trend was also supported by the scRNA-seq dataset GSE207651 (Figure 5E). Within the single-cell dataset, Prtn3 expression was predominantly enriched in monocyte clusters (Figures 5F,G). These results suggest that monocytes are a major cellular source of Prtn3 upregulation in SLI.

### Choline suppresses monocyte inflammatory activation and migration

**FIGURE 6:**
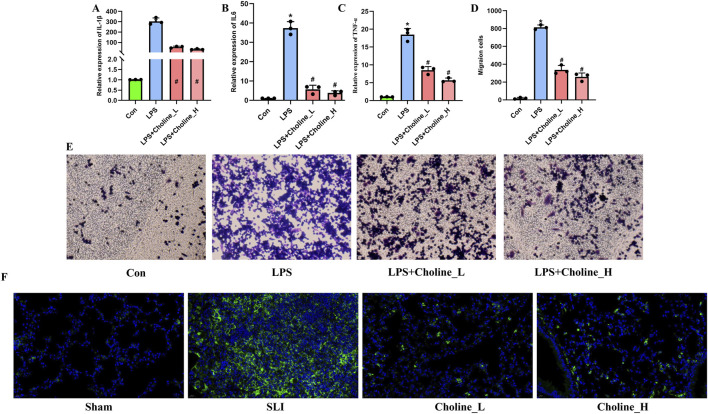
Effects of choline on monocyte activation and migration. **(A–C)** Relative mRNA expression levels of IL6, TNF-α, and IL-1β in THP-1 cells. **(D,E)** Transwell migration assay of THP-1 cells with crystal violet staining. **(F)** Representative immunofluorescence images of F4/80 in lung tissue. n = 3 for each group. Data are presented as mean ± SD. *P < 0.05 vs. control or Sham group; #P < 0.05 vs. LPS or CLP group.

Because single-cell analysis suggested that Prtn3 is predominantly expressed in monocytes, we next examined whether choline affects monocyte activation. THP-1 cells were stimulated with LPS in the presence or absence of choline, and inflammatory cytokine expression was assessed. LPS stimulation markedly increased the expression of IL-1β, IL-6, and TNF-α, whereas choline treatment significantly reduced the expression of these cytokines (Figures 6A–C). To determine whether choline influences monocyte migratory behavior, Transwell assays were performed. LPS stimulation significantly enhanced monocyte migration, whereas choline treatment reduced LPS-induced transmigration (Figures 6D,E). These findings suggest that choline suppresses inflammatory activation and migratory responses in monocytes. To validate these observations *in vivo*, lung sections were stained for F4/80. The number of F4/80-positive cells was increased in CLP-treated mice and was reduced after choline treatment (Figure 6F), supporting the notion that choline attenuates inflammatory monocyte/macrophage accumulation in lung tissue.

### Prtn3 overexpression partially reverses the inhibitory effects of choline on monocyte activation via NF-κB signaling

**FIGURE 7:**
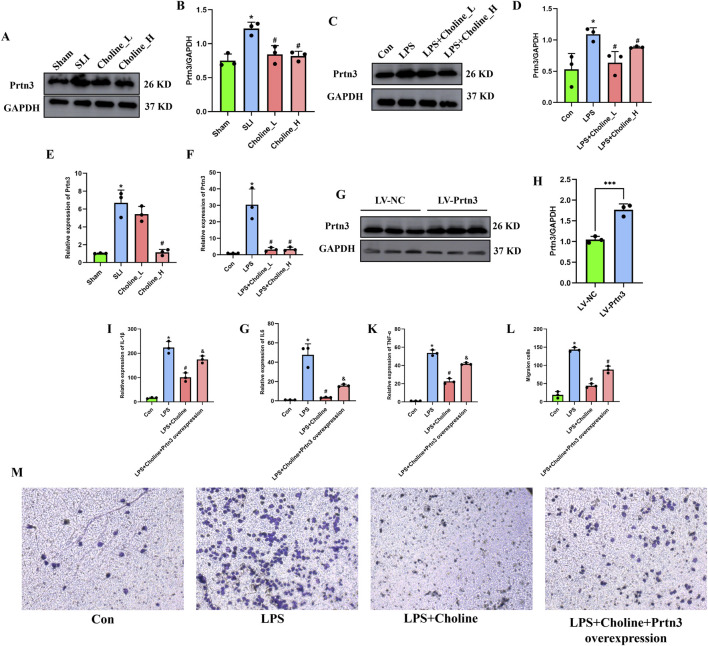
Effects of choline on monocyte activation via Prtn3. **(A–D)** Protein expression of Prtn3 in lung tissues **(A,B)** and THP-1 cells **(C,D)**. **(E,F)** mRNA expression of Prtn3 in lung tissues **(E)** and THP-1 cells **(F)**. **(G,H)** Validation of Prtn3 overexpression in THP-1 cells. **(I–K)** Relative mRNA expression levels of IL6, TNF-α, and IL-1β. **(L,M)** Transwell migration assay of THP-1 cells. n = 3 for each group. Data are presented as mean ± SD. *P < 0.05 vs. control; #P < 0.05 vs. LPS or CLP group.

Prtn3 expression was first assessed in THP-1 cells and lung tissue to define its relationship with choline treatment. RT-qPCR and Western blotting revealed marked Prtn3 upregulation following LPS stimulation in THP-1 cells and CLP challenge in mouse lung tissue, whereas choline treatment significantly reduced Prtn3 mRNA and protein levels in both models (Figures 7A–F). We next generated THP-1 cells with stable lentiviral-mediated Prtn3 overexpression, which was confirmed by Western blotting (Figures 7G,H). To further explore the upstream cholinergic mechanism by which choline regulates Prtn3, we analyzed the expression profile of cholinergic receptors in monocytes using single-cell RNA-seq data. Among the examined cholinergic receptor-related genes, Chrm3 showed the highest average expression in monocytes, suggesting that CHRM3 may be involved in choline-mediated regulation of monocyte responses (Supplementary File 2; Supplementary Figure S5A). We therefore treated THP-1 cells with tiotropium, a muscarinic receptor antagonist with inhibitory activity against CHRM3. Notably, tiotropium significantly reversed the inhibitory effect of choline on Prtn3 expression under LPS-stimulated conditions, as evidenced by increased Prtn3 mRNA levels in the LPS + choline + tiotropium group compared with the LPS + choline group (Supplementary File 2; Supplementary Figure S5B). These findings suggest that choline suppresses Prtn3 expression, at least in part, through CHRM3-dependent cholinergic signaling.

**FIGURE 8:**
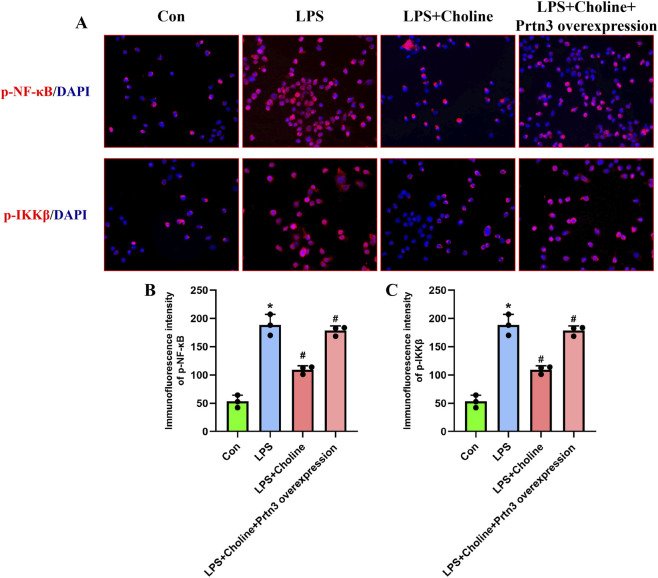
Prtn3 overexpression reverses the inhibitory effects of choline on NF-κB signaling in THP-1 cells. **(A)** Immunofluorescence staining of p-NF-κB and p-IKKβ in THP-1 cells under different treatments. Nuclei were counterstained with DAPI. **(B,C)** Quantitative analysis of the fluorescence intensity of p-NF-κB **(B)** and p-IKKβ **(C)**. n = 3 for each group. Data are presented as mean ± SD. *P < 0.05 vs. control; #P < 0.05 vs. LPS group.

Functionally, Prtn3 overexpression enhanced monocyte migration and restored pro-inflammatory cytokine expression compared with the LPS + choline group (Figures 7I–M). Because NF-κB signaling is a central driver of sepsis-associated monocyte/macrophage activation and cytokine production, and cholinergic anti-inflammatory signaling has been shown to restrain inflammation partly through NF-κB inhibition (Mussbacher et al., 2023; Liu et al., 2018; Parrish et al., 2008), we examined whether Prtn3 counteracts the suppressive effects of choline via this pathway. Immunofluorescence staining showed that LPS markedly increased p-NF-κB and p-IKKβ fluorescence intensity in THP-1 cells, indicating robust NF-κB pathway activation (Figures 8A–C). Choline substantially attenuated NF-κB and IKKβ phosphorylation, consistent with suppression of LPS-induced inflammatory signaling. This inhibitory effect was largely reversed by Prtn3 overexpression, as reflected by restored p-NF-κB and p-IKKβ signals. Collectively, these results suggest that Prtn3 promotes monocyte activation through NF-κB signaling, whereas choline mitigates inflammatory responses, at least in part, by suppressing Prtn3-dependent NF-κB activation.

**FIGURE 9:**
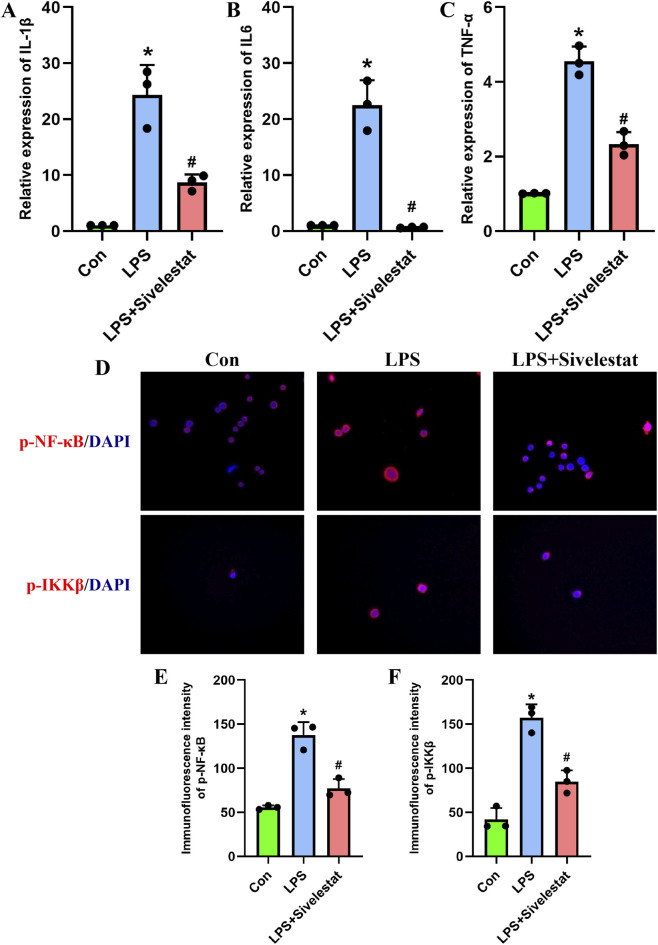
Prtn3 inhibition suppresses inflammatory cytokine expression and NF-κB signaling in LPS-stimulated THP-1 cells. **(A–C)** Relative mRNA expression levels of IL-1β, IL6, and TNF-α in THP-1 cells. **(D)** Immunofluorescence staining of p-NF-κB and p-IKKβ. **(E,F)** Quantitative analysis of the fluorescence intensity of p-NF-κB **(E)** and p-IKKβ **(F)**. n = 3 for each group. Data are presented as mean ± SD. *P < 0.05 vs. control; #P < 0.05 vs. LPS group.

Building on the Prtn3 overexpression results, we next asked whether pharmacological inhibition of Prtn3 could conversely restrain monocyte inflammatory activation. Under LPS-stimulated conditions, treatment with the Prtn3 inhibitor sivelestat markedly reduced IL-1β, IL-6, and TNF-α expression (Figures 9A–C). Consistently, sivelestat substantially attenuated the phosphorylation-associated fluorescence signals of NF-κB and IKKβ, indicating suppression of the NF-κB signaling cascade (Figures 9D–F). These loss-of-function data complement the Prtn3 overexpression findings and further support Prtn3 as a positive regulator of monocyte inflammatory activation through the NF-κB pathway.

## Discussion

Sepsis is a life-threatening syndrome characterized by infection-induced organ dysfunction resulting from a dysregulated host response (Rhodes et al., 2017). Increasing evidence suggests that metabolic alterations contribute to the progression of sepsis and that restoring metabolic balance may represent a potential therapeutic strategy (Liu et al., 2022). In the present study, we observed that serum choline levels were significantly decreased in CLP-induced SLI mice. This finding suggest that choline deficiency may be associated with sepsis and its related organ injury. Based on this observation, we investigated whether choline supplementation could exert protective effects in sepsis. Our results demonstrated that choline administration improved survival, reduced systemic inflammation, and alleviated sepsis-associated symptoms in CLP-treated mice.

Excessive inflammation is a major driver of early organ injury in sepsis, particularly in the lung, where it contributes to the development of SLI. In this study, choline supplementation markedly attenuated lung injury, as evidenced by reduced pulmonary edema, inflammatory cell infiltration, and pro-inflammatory cytokine production. These findings indicate that choline exerts anti-inflammatory effects in the context of SLI. Previous studies have reported that choline can modulate inflammatory responses in different pathological conditions. For example, choline has been shown to aggravate H. pylori-induced inflammation (Li S. et al., 2020), while other studies suggest that it can alleviate neuroinflammation and oxidative stress under specific conditions (Huang et al., 2025). However, its effects on SLI have not been well characterized. Our study extends these observations by demonstrating a protective role of choline in a clinically relevant SLI model.

To further elucidate the mechanisms underlying the protective effects of choline in SLI, we integrated transcriptomic and proteomic datasets and identified a subset of overlapping candidates whose dysregulated expression in SLI was partially reversed by choline treatment. Among these molecules, Prtn3 was prioritized because PPI analysis revealed the highest network connectivity for Prtn3, suggesting that it may act as a hub molecule in the molecular response to choline in SLI. Prtn3, also known as proteinase 3, is a serine protease predominantly expressed in myeloid-derived cells, particularly neutrophils and monocytes (Csernok et al., 1994; Alatrash et al., 2012; Mayet et al., 1993). Emerging evidence has implicated Prtn3 in sepsis-associated inflammatory injury, including vascular dysfunction, endothelial barrier disruption, and excessive inflammatory responses (Patterson et al., 2021; Zhang, 2024). To further clarify its potential role in SLI, we firstly examined the cellular distribution of Prtn3 using single-cell RNA-seq. This analysis showed that Prtn3 was predominantly expressed in monocytes, suggesting that these cells may represent an important cellular source of Prtn3 in SLI. Given the established role of monocytes/macrophages in amplifying inflammation and tissue damage during septic lung injury (Huang et al., 2023), and Prtn3 has been linked to monocyte-to-macrophage differentiation (Cheung et al., 2021), these findings support a potential link between Prtn3 and monocyte-associated inflammatory responses. Consistently, we observed that Prtn3 expression was significantly increased in LPS-activated THP-1 monocytes and in the lung tissue of SLI mice, whereas choline treatment markedly reduced Prtn3 levels in both contexts, highlighting its regulatory potential. These findings provide a rationale to investigate the effects of choline on Prtn3 regulation and monocyte-mediated inflammatory responses.

However, whether Prtn3 directly mediates the effects of choline on monocyte function remains unclear. To address this, we performed functional assays to evaluate monocyte activation and migration in the presence of choline and manipulated Prtn3 expression. Our data indicate that choline suppresses monocyte activation and migration both *in vitro* and *in vivo*. Importantly, overexpression of Prtn3 partially reversed the inhibitory effects of choline on LPS-induced monocyte activation, including cytokine production and cell migration. These gain-of-function findings suggest that Prtn3 is not merely a choline-responsive molecule but also a functional mediator capable of sustaining monocyte inflammatory activation under choline-treated conditions.

Mechanistically, our data further link Prtn3 to NF-κB signaling, a canonical pathway governing sepsis-associated monocyte/macrophage activation and inflammatory cytokine production. Choline markedly reduced the phosphorylation-associated fluorescence signals of NF-κB and IKKβ in LPS-stimulated THP-1 cells, whereas Prtn3 overexpression largely restored these signals despite choline treatment. This observation indicates that Prtn3 can counteract the inhibitory effect of choline on the IKKβ/NF-κB axis. Conversely, pharmacological inhibition of Prtn3 with sivelestat suppressed IL-1β, IL-6, and TNF-α expression and attenuated NF-κB and IKKβ phosphorylation under LPS-stimulated conditions. Together, these complementary gain- and loss-of-function data strengthen the conclusion that Prtn3 acts as a positive regulator of monocyte inflammatory activation, at least in part by sustaining NF-κB pathway activation. They also provide a mechanistic explanation for how choline may restrain monocyte-driven inflammation in SLI through suppression of Prtn3-dependent NF-κB signaling.

Despite these findings, several limitations should be acknowledged. First, choline was administered shortly after CLP induction, which may not fully reflect clinical treatment scenarios. Future studies should evaluate different dosing regimens and therapeutic time windows. Second, although two doses of choline were evaluated in this study, the dose interval between the low-dose and high-dose groups was relatively narrow, which limited our ability to establish a definitive dose-response relationship or define the optimal therapeutic window. Third, although tiotropium and sivelestat were used to pharmacologically inhibit CHRM3-related cholinergic signaling and Prtn3 activity, respectively, potential off-target effects cannot be fully excluded. Future studies using CHRM3 knockdown/overexpression models, Prtn3-deficient mice, or monocyte-specific Prtn3 knockdown models would provide more definitive evidence for the CHRM3–Prtn3 axis in choline-mediated protection against SLI. Additionally, although lung injury was assessed using histology, BALF protein, W/D ratio, and cytokine measurements, direct lung function and quantitative morphometric analyses were not performed, which should be addressed in future studies to strengthen translational relevance. Finally, our *in vitro* experiments were mainly performed in THP-1 cells, which may not fully reflect primary monocyte/macrophage biology. Future studies using primary monocytes/macrophages are needed to validate these findings.

In conclusion, our study demonstrates that choline supplementation attenuates sepsis-induced lung injury and systemic inflammation, potentially through modulation of monocyte activation and Prtn3 expression. Mechanistically, choline appears to suppress Prtn3-dependent NF-κB signaling, thereby limiting monocyte inflammatory activation. These findings provide new insights into the role of choline in sepsis and suggest that targeting the choline–Prtn3–NF-κB axis may represent a promising therapeutic strategy.

## Data Availability

The original contributions presented in the study are included in the article/Supplementary Material, further inquiries can be directed to the corresponding author.
