# Supplementary material for: Choline supplementation protects against sepsis-induced lung injury, potentially through suppression of Prtn3-associated monocyte activation
Source: Front Pharmacol. 2026 Jun 18;17:1839871. doi: 10.3389/fphar.2026.1839871 (PMC13322800; doi:10.3389/fphar.2026.1839871)
Supplement: Supplementary file 2 [file Supplementaryfile2.docx]

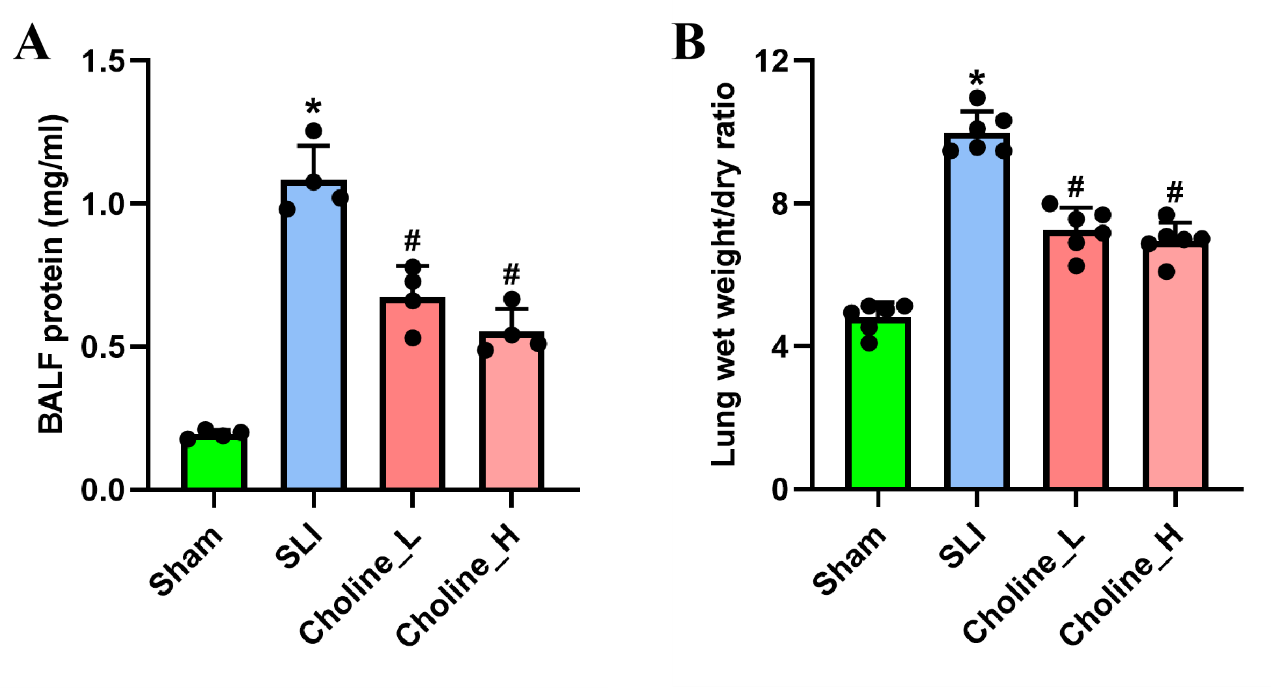
**Fig S1 Choline supplementation reduces alveolar-capillary barrier permeability and pulmonary edema in SLI mice.** (A) Protein concentration in bronchoalveolar lavage fluid (BALF). (B) Lung wet-to-dry weight ratio. n = 5 for each group. Data are presented as mean ± SD. *P < 0.05 vs Sham group; #P < 0.05 vs SLI group.


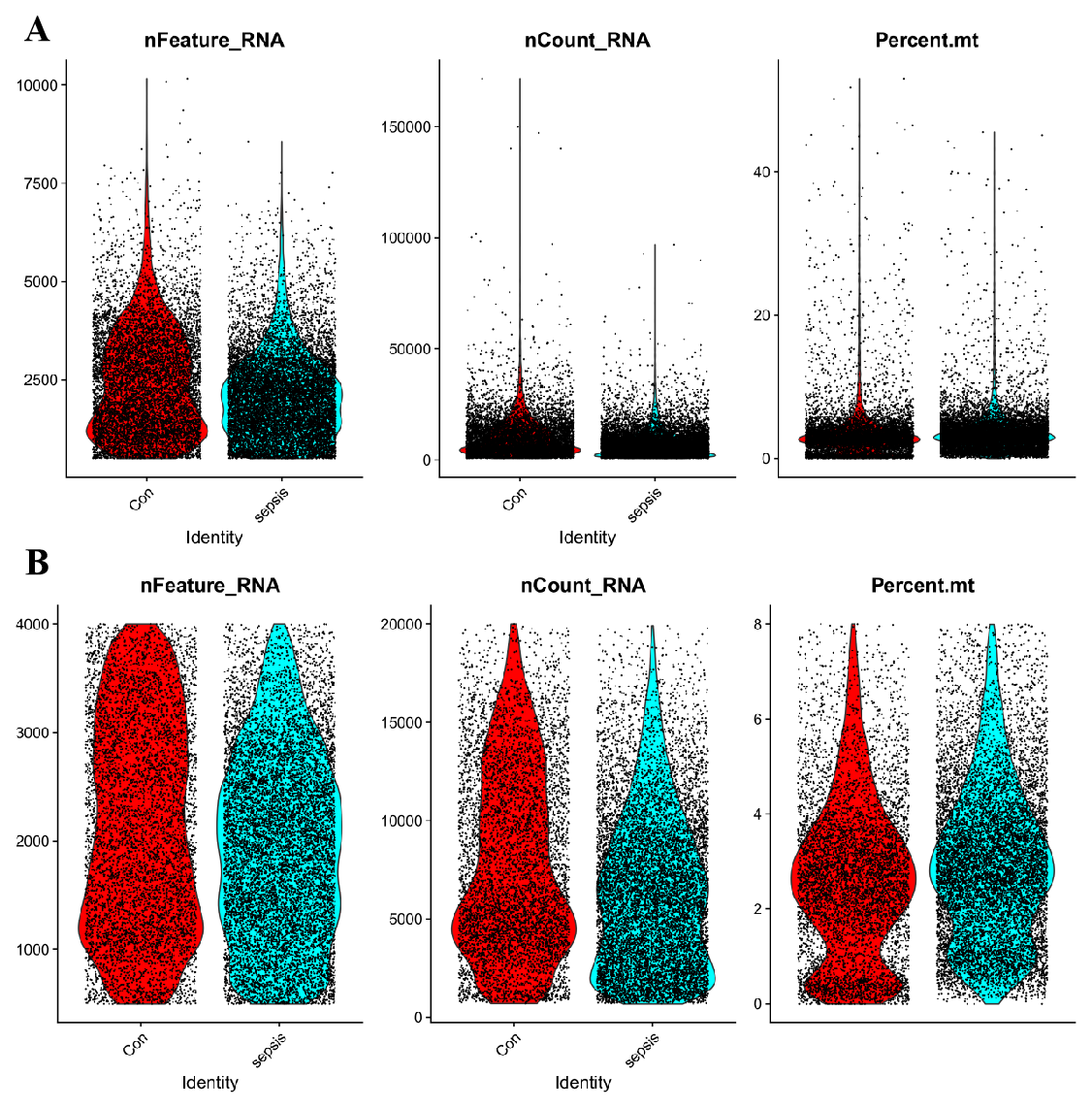


**Fig S2** Quality control (QC) of scRNA-seq data. (A) Before QC; (B) After QC.


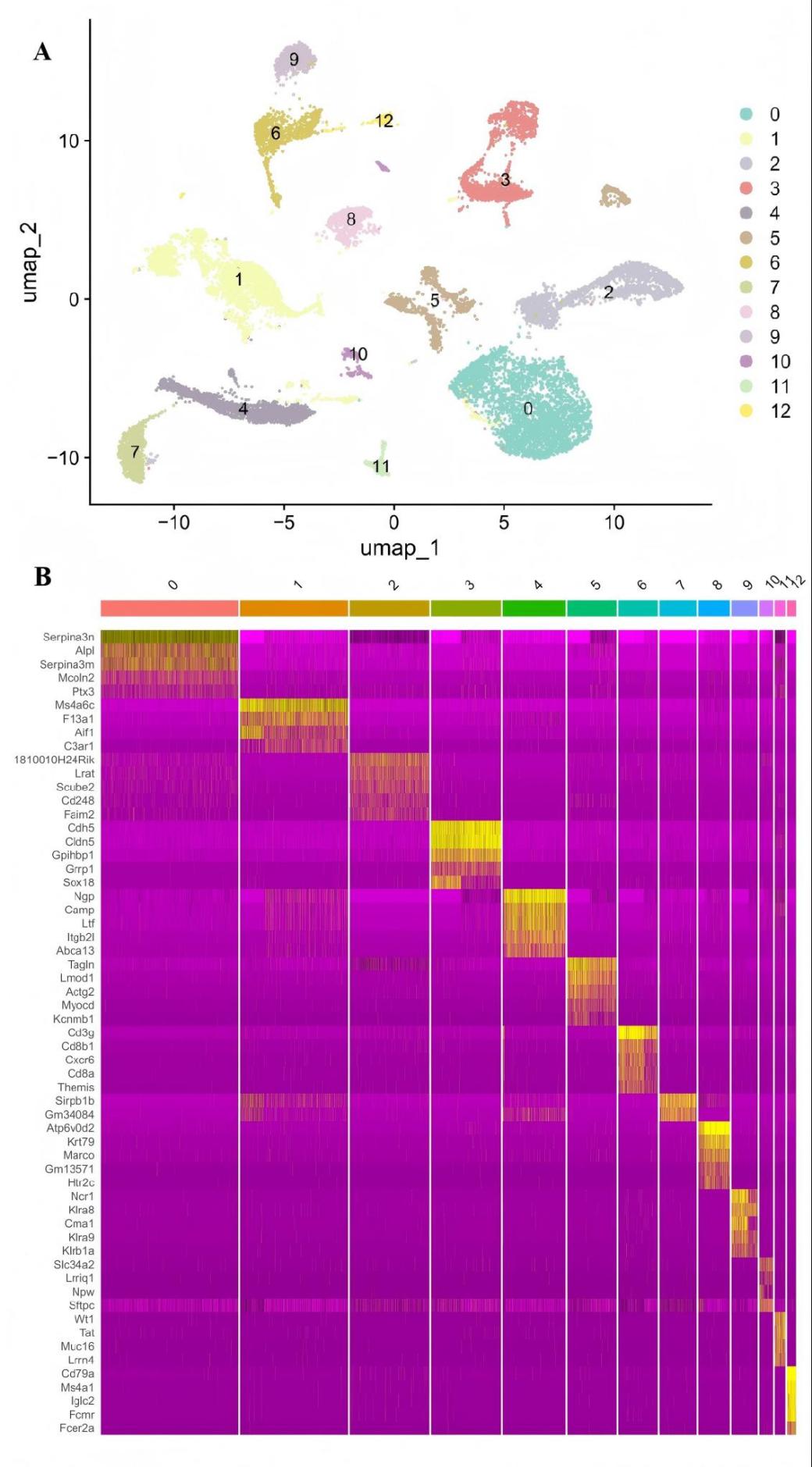


**Fig S3** UMAP for scRNA-seq data set GSE207651 before cell population annotation, and heatmap of differentially expressed markers in different cell population.


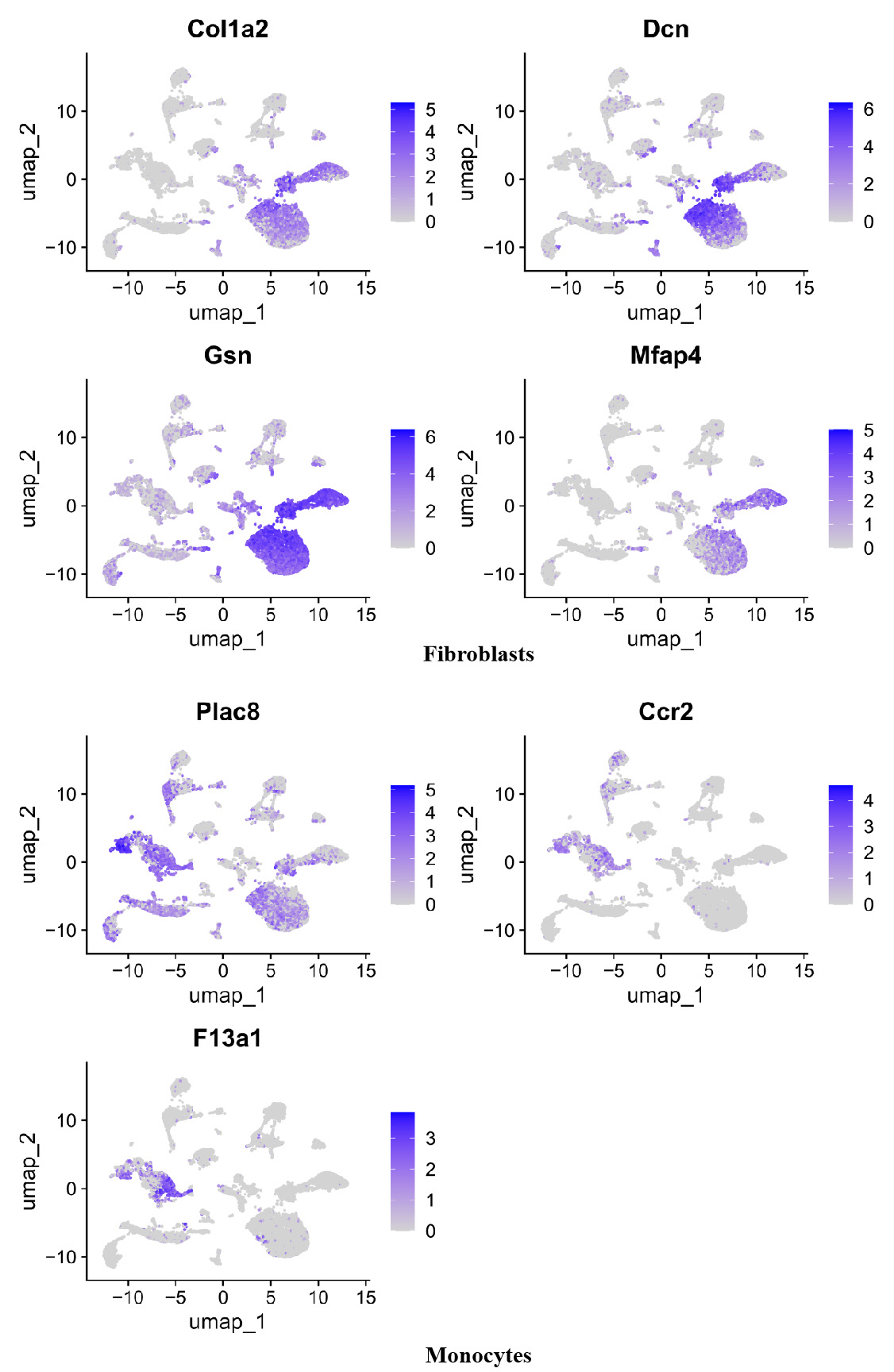


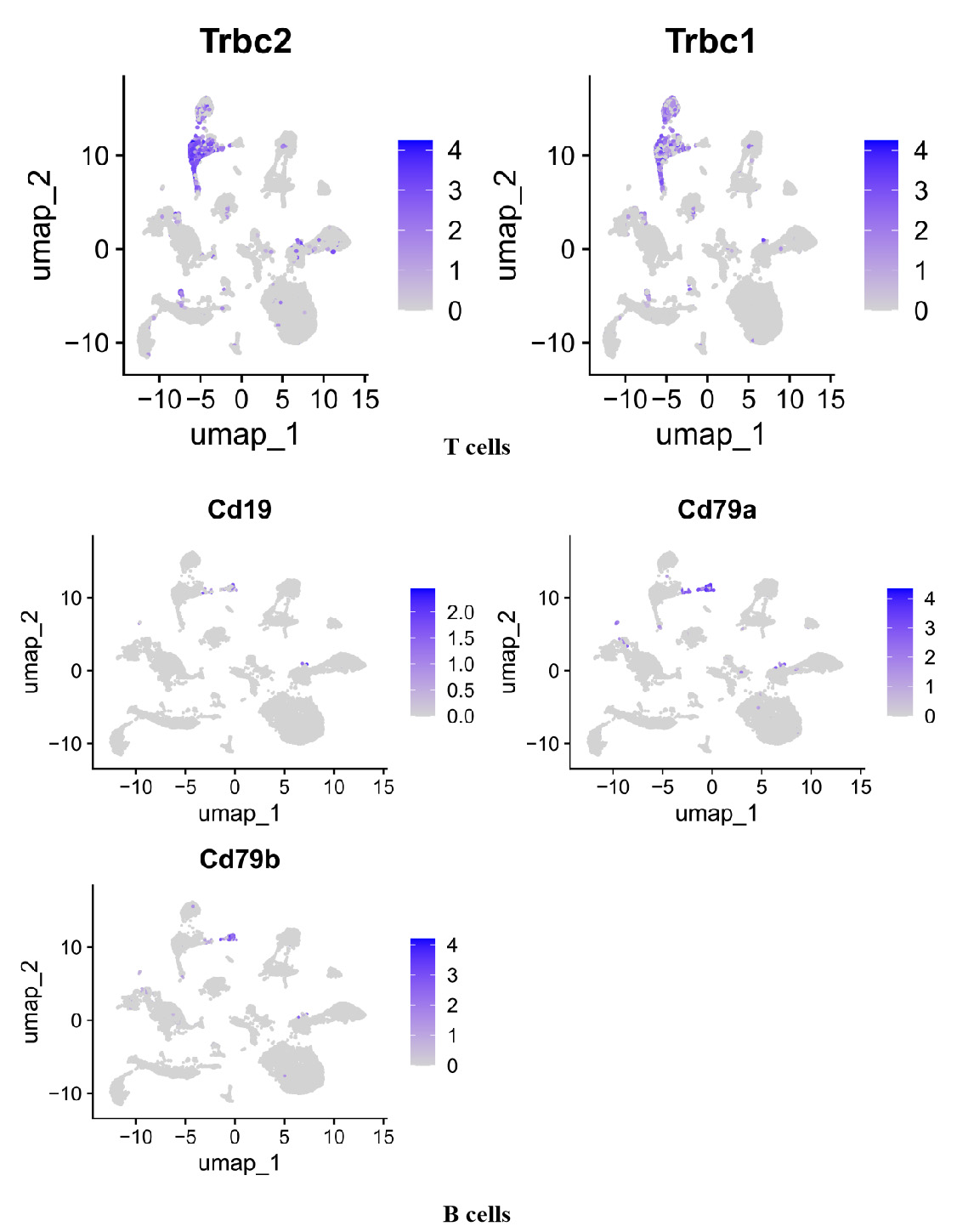


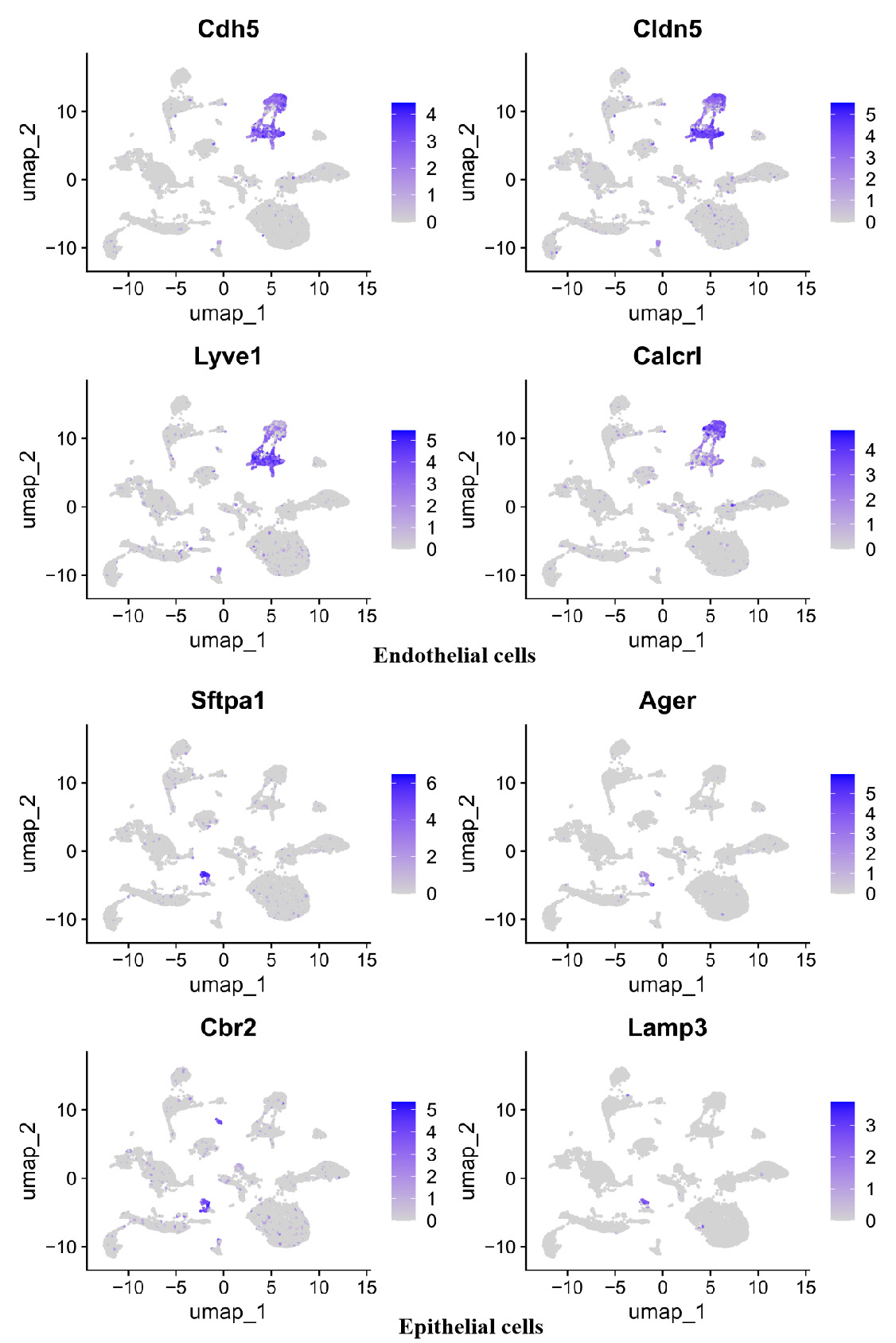


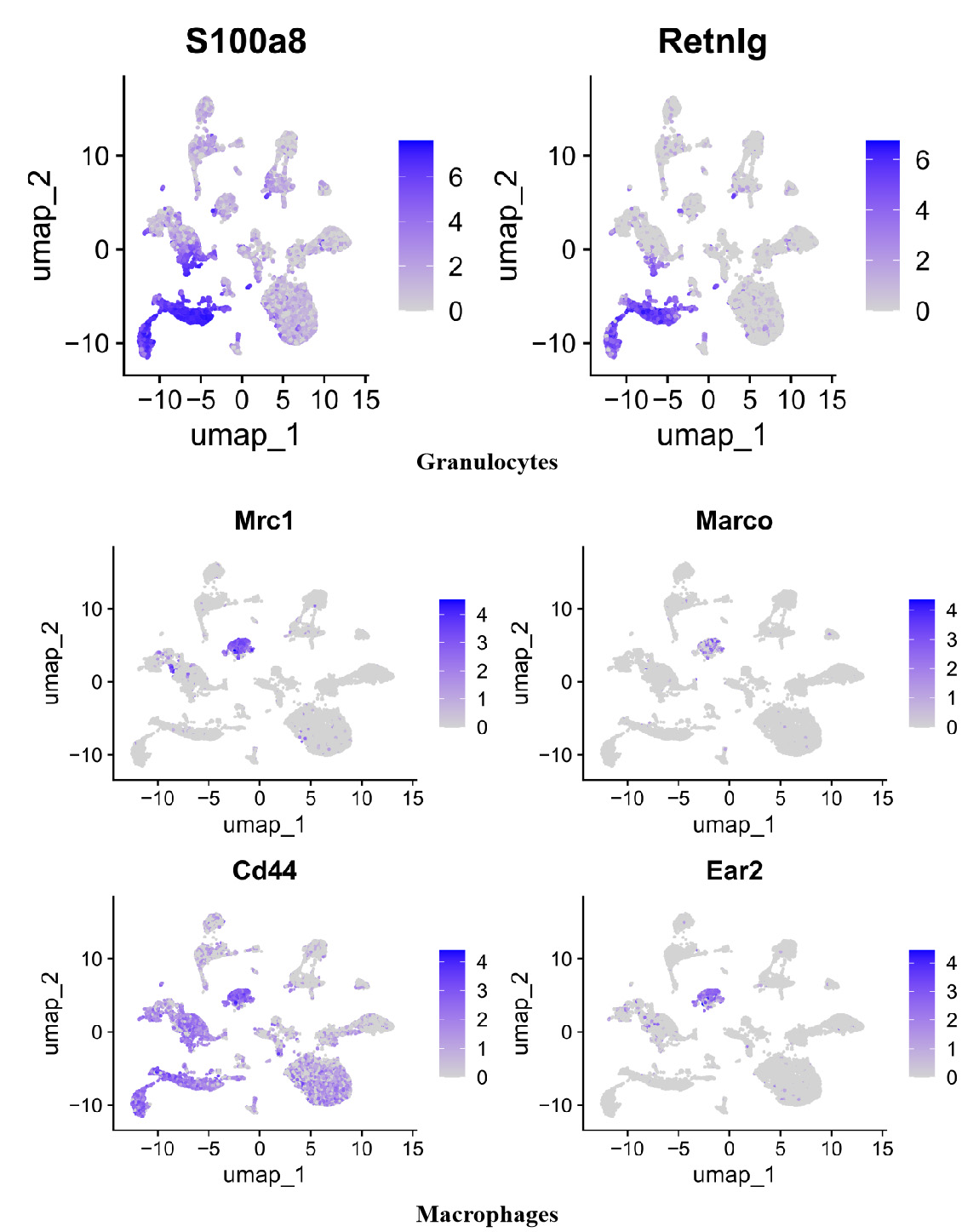


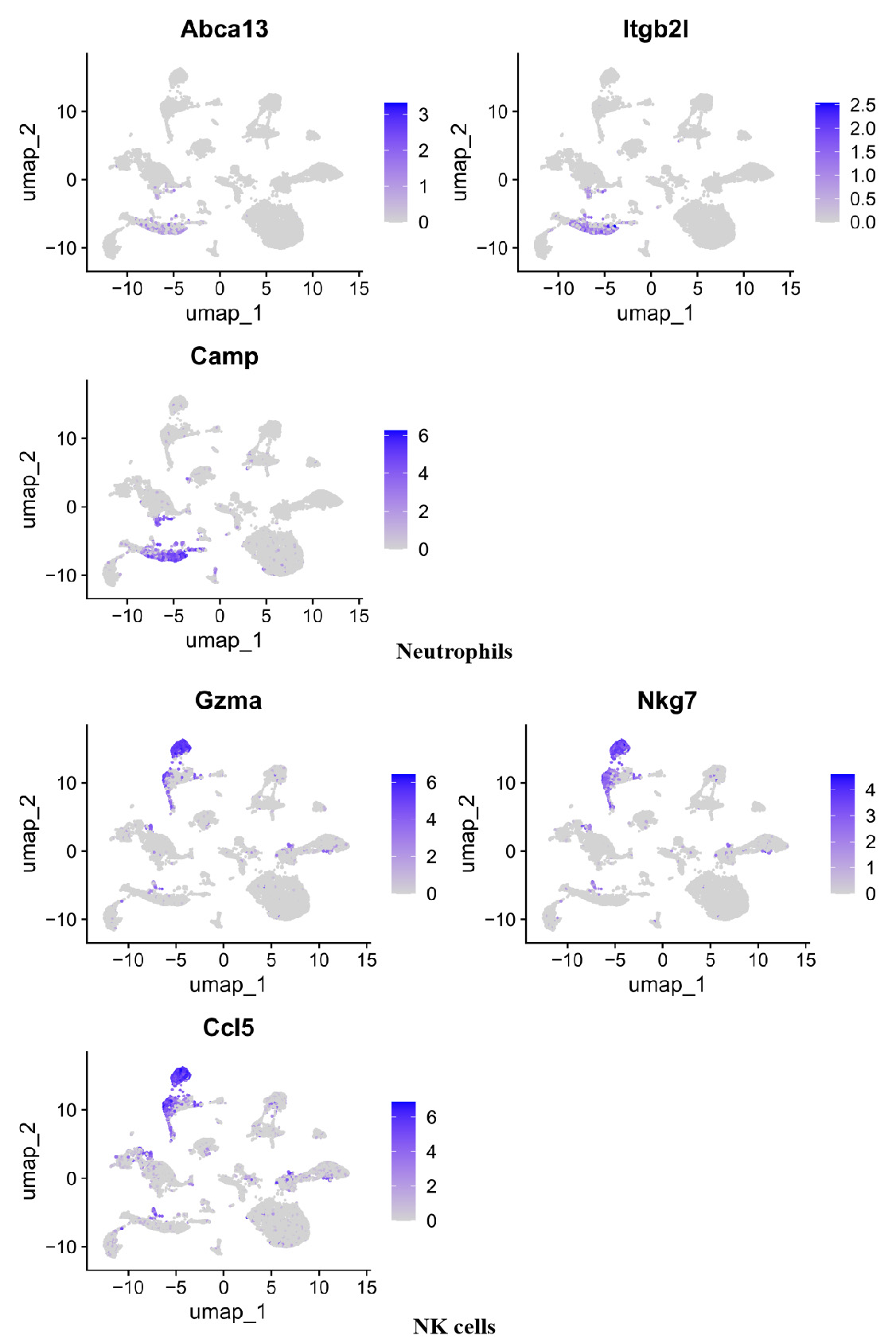


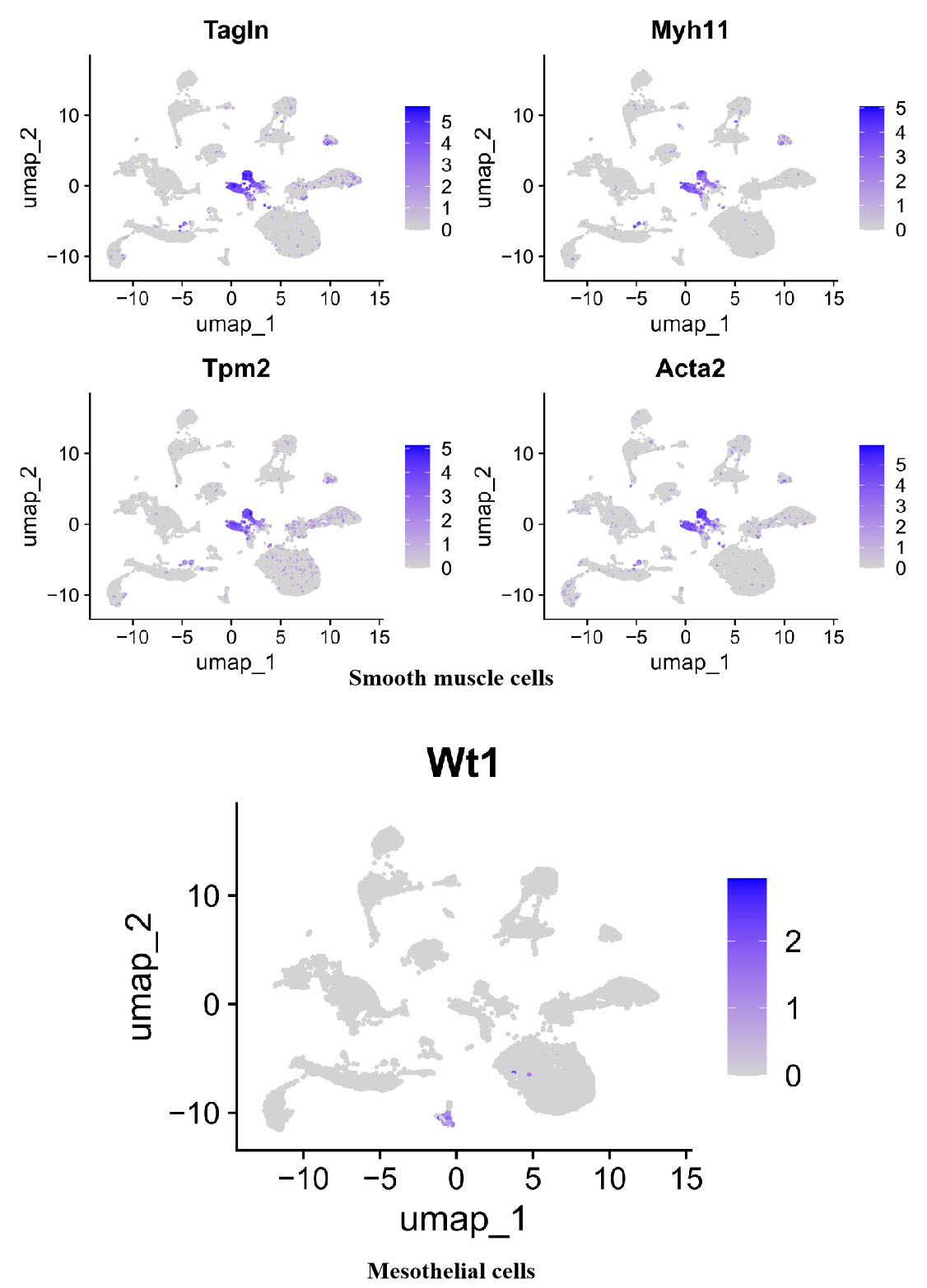


**Fig S4** UMAP of different cell population signatures used for annotation.


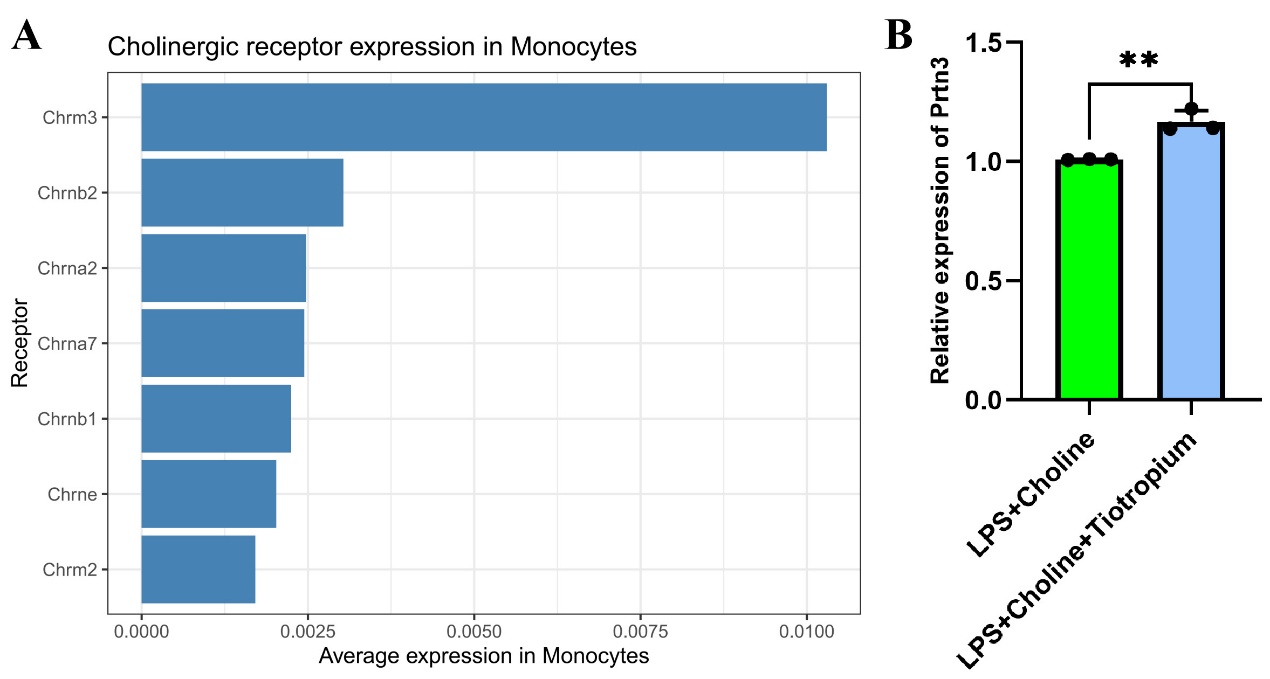


**Fig S5** CHRM3 mediates the inhibitory effect of choline on Prtn3 expression in monocytes.
(A) Average expression levels of cholinergic receptor-related genes in monocytes based on single-cell RNA-seq analysis. (B) Relative mRNA expression of Prtn3 in THP-1 cells treated with tiotropium under LPS and choline stimulation. n = 3 for each group. Data are presented as mean ± SD. **P < 0.01 vs LPS + choline group.
